# A comparison of efficacy of six prediction models for intravenous immunoglobulin resistance in Kawasaki disease

**DOI:** 10.1186/s13052-018-0475-z

**Published:** 2018-03-09

**Authors:** Weiguo Qian, Yunjia Tang, Wenhua Yan, Ling Sun, Haitao Lv

**Affiliations:** Department of Cardiology, Children’s Hospital of Soochow University, Suzhou, China

**Keywords:** Kawasaki disease, Children, Prediction model, Intravenous immunoglobulin resistance

## Abstract

**Background:**

Kawasaki disease (KD) is the most common pediatric vasculitis. Several models have been established to predict intravenous immunoglobulin (IVIG) resistance. The present study was aimed to evaluate the efficacy of prediction models using the medical data of KD patients.

**Methods:**

We collected the medical records of patients hospitalized in the Department of Cardiology in Children’s Hospital of Soochow University with a diagnosis of KD from Jan 2015 to Dec 2016. IVIG resistance was defined as recrudescent or persistent fever ≥36 h after the end of their IVIG infusion.

**Results:**

Patients with IVIG resistance tended to be younger, have higher occurrence of rash and changes of extremities. They had higher levels of c-reactive protein, aspartate aminotransferase, neutrophils proportion (N%), total bilirubin and lower level of albumin. Our prediction model had a sensitivity of 0.72 and a specificity of 0.75. Sensitivity of Kobayashi, Egami, Kawamura, Sano and Formosa were 0.72, 0.44, 0.48, 0.20, and 0.68, respectively. Specificity of these models were 0.62, 0.82, 0.66, 0.91, and 0.48, respectively.

**Conclusions:**

Our prediction model had a powerful predictive value in this area, followed by Kobayashi model while all the other prediction models had less excellent performances than ours.

## Background

Kawasaki disease (KD) is a systemic self-limited vasculitis that mostly occurs in children under 5 years of age. The main complication of the disease is coronary artery abnormality (CAA), which is considered the leading cause of acquired heart disease [1]. Fifty years have passed since the first report of the disease and now 2 g/kg intravenous immunoglobulin (IVIG) together with large amount of aspirin is considered the golden primary treatment in the acute stage worldwide. However, approximately 10–20% patients do not respond to such treatment and presented with prolonged changes in presence of prolonged fever [1]. Moreover, alternative treatments after response failure to initial IVIG treatment are usually unsatisfactory, with a remarkable higher incidence of CAA [2, 3]. Thus, early identification of IVIG resistance is of great importance so that additional therapy in the early stage could be given to reduce the risk of CAA.

In 2006, Kobayashi established a prediction score model of IVIG resistance based on 676 study patients [4]. The related predictors were serum sodium (Na) ≤ 133 mmol/L, days of illness at initial treatment ≤4, aspartate aminotransferase (AST) ≥ 100 IU/L, N% ≥ 80%, c-reactive protein (CRP) ≥ 10 mg/dL, age ≤ 12 months and platelet count (PLT) ≤ 30 × 10^4^/mm^3^. Due to the racial heterogeneity, studies on IVIG resistance prediction score have amassed in recently years in different countries and areas [2, 5–8].

Previously we have established a prediction model using the KD dataset in this area from 2006 to 2014 [9]. In the present study, we aimed to evaluate the efficacy of these prediction models using the medical data of KD patients hospitalized in Children’s Hospital of Soochow University during the last 2 years.

## Methods

We prospectively collected the medical records of patients hospitalized in the Department of Cardiology in Children’s Hospital of Soochow University with a diagnosis of KD from Jan 2015 to Dec 2016. When we evaluated the Kawamura prediction model and parts of the Formosa model, the medical records were retrospectively collected. Children’s Hospital of Soochow University is a tertiary hospital that serves most children of this area and some of the children from surrounding cities. Kawasaki disease was confirmed when patients had fever for more than 5 days plus four of the following five characteristics. 1. Rash. 2. Bilateral conjunctive injection. 3. Cervical lymphadenopathy. 4. Changes of the extremities. 5. Oral mucosal changes. Incomplete KD was defined as patients with ≥5 days of fever and < 4 classic criteria, but who had CAA upon echocardiography or a set of suspect laboratory criteria according to the AHA guideline [10]. IVIG resistance was referred to when a patient developed recrudescent or persistent fever ≥36 h after the end of their IVIG infusion [10]. All febrile patients received 2 g/kg IVIG and 30-50 mg/kg aspirin as the guideline recommended [10]. The dosage of aspirin was decreased to 3–5 mg/kg/day until the patients were afebrile for 3–4 days. All parents of the patients gave their informed consent. The study was approved by the Ethics Committee of Children’s Hospital of Soochow University (No: 2014LW003).

Demographic and clinical characteristics were obtained on admission. Laboratory data regarding CRP, PLT, Na, N%, serum albumin (ALB), alanine aminotransferase (ALT), AST, total bilirubin (TB), neutrophils count, lymphocytes count were obtained within 24 h on admission. In our prediction score model, two points were assigned to age younger than 6 months and ALB lower than 35 g/L. One point was assigned to N% ≥80%, presence of rash and edema of extremities. Our model had a sensitivity of 71.4% and a specificity of 76.0%. Detailed information of the prediction models are shown in Table 1.

**Table 1:** Prediction score models of IVIG resistance in KD patients

Prediction models	Nation	Year	Enrolled patients, n	Patients with IVIG resistance, n	Sensitivity, %	Specificity, %	Risk factors	Points	Predicted risk (score)
Kobayashi	Japan	2006	528	148	86	68	Age ≤ 12 mo	1	Low risk (0–2) High risk (≥ 3)
							Illness days ≤4 d	2	
							CRP ≥ 10 mg/dL	1	
							AST ≥ 100 IU/L	2	
							PLT ≤ 300 × 10^9^/L	1	
							Na ≤ 133 mmol/L	2	
							N% ≥ 80%	2	
Egami	Japan	2006	320	41	78	76	Age ≤ 6 mo	1	Low risk (0–2) High risk (≥ 3)
							Illness days ≤4 d	1	
							CRP ≥ 8 mg/dl	1	
							ALT ≥80 IU/L	2	
							PLT < 300 × 10^9^/L	1	
Kawamura	Japan	2016	405	85	71	69	NLR ≥ 3.83 PLR ≥ 150	- -	–
Sano	Japan	2007	112	22	77	86	CRP ≥ 7.0 mg	1	Low risk (0–1) High risk (≥ 2)
							TB ≥ 0.9 mg/dl	1	
							AST ≥ 200 IU/L	1	
Formosa	Taiwan	2015	248	29	86.2	81.3	lymphadenopathy	1	Low risk (0–2)
							N% ≥ 60%	2	High risk (≥ 3)
							ALB < 35 g/L	1	
Ours	China	2016	910	46	71.4	76.0	Age < 6 months	2	Low risk (0–2) High risk (≥ 3)
							Rash	1	
							Edema of extremities	1	
							N% ≥80%	1	
							ALB < 35 g/L	2	

*CRP* c-reactive protein, *PLT* platelet count, *Na* serum sodium, *N%* percentage of neutrophils, *ALB* serum albumin, *ALT* alanine aminotransferase, *AST* aspartate aminotransferase, *TB* total bilirubin, *NLR* neutrophil-to-lymphocyte ratio, *PLR* platelet-to-lymphocyte ratio

### Statistical analysis

All analyses were carried out by means of a SPSS statistical software package, version 22.0 (IBM, Armonk, NY, USA). Data are expressed as mean ± standard deviation (SD), median with quartiles or number with percentage as appropriate. Descriptive statistics were performed on the demographic characteristics. Parametric and nonparametric comparative tests for continuous data and χ^2^ test for categorical data were used to compare variables between groups. Sensitivity, specificity, positive predictive value (PPV) and negative predictive value (NPV) were used to determine the efficacy of the prediction models. Area under the receiver operating characteristic (ROC) curve was calculated to evaluate capacity of each model. A *P* < 0.05 was considered statistically significant.

## Results

A total of 505 patients were diagnosed with KD during the study period. One patient failed to be treated with IVIG for her parents’ refusal. As a result, 504 patients were enrolled in the present study. 318 were males and 186 were females, yielding a male-to-female ratio of 1.71:1. 153 (30.3%) patients were under one year old and 467 (92.7%) were under five years old. The median age were 20 (11, 36) months. 322 (63.9%) patients were complete KD while the remaining 182 (36.1%) were incomplete cases. 25 (5.0%) patients were identified as IVIG resistance. The characteristics of the patients are shown in Table 2. Significant differences were demonstrated in age <  6 months, presence of rash and edema of extremities. For laboratory findings, patients with IVIG resistance had higher levels of CRP, AST, N% and TB. On the other hand, they tended to have lower level of ALB.

**Table 2:** Demographic, clinical and laboratory characteristics between intravenous immunoglobulin (IVIG) resistant and IVIG responsive KD patients

Variables	Total (*n* = 504)	IVIG resistant patients (*n* = 479)	IVIG responsive patients (*n* = 25)	*P* value
Male, *n* (%)	318 (63.1)	303 (60.1)	15 (3.0)	0.726
Age in months, median (quartile)	20 (11, 36)	20 (11, 35)	15 (5.5, 42.5)	0.456
< 1 yr., *n* (%)	153 (30.4)	142 (28.2)	11 (2.2)	0.128
< 6 months, *n* (%)	58 (11.5)	51 (10.1)	7 (1.4)	0.008
Oral mucosal changes, *n* (%)	457 (90.7)	435 (86.3)	22 (4.4)	0.720^*^
Strawberry tongue, *n* (%)	384 (76.2)	367 (72.8)	16 (3.2)	0.150
Rash, *n* (%)	394 (78.2)	370 (73.4)	24 (4.8)	0.027
Edema of extremities, *n* (%)	224 (44.4)	204 (40.5)	20 (4.0)	< 0.001
Desquamation of the fingertips, *n* (%)	268 (53.2)	252 (50.0)	16 (3.2)	0.266
Cervical lymphadenopathy, *n* (%)	398 (79.0)	380 (79.4)	18 (3.6)	0.381
Days of illness at initial treatment, mean ± SD (median), days	6.8 ± 2.0 (6.0)	6.8 ± 2.0 (6.0)	6.2 ± 2.0 (6.0)	0.156
CRP, median (quartile), mg/dl	69.1 (37.6, 112.7)	65.6 (36.3, 111.4)	94.4 (84.2, 134.9)	0.001
ALT, median (quartile), IU/L	22.7 (13.4, 55.7)	22.2 (13.2, 54.1)	29.5 (17.8, 104.5)	0.051
AST, median (quartile), IU/L	31.0 (24.3, 43.8)	31.0 (24.1, 43.0)	42.6 (28.5, 53.6)	0.034
Na, mean ± SD (median), mmol/L	135 (133, 137)	135 (133, 137)	133 (131, 137)	0.247
ALB, mean ± SD (median), g/L	38.6 (35.8, 40.7)	38.8 (35.8, 40.8)	36.3 (32.8, 38.8)	0.005
PLT, mean ± SD (median), × 10^9^/L	357 (284, 453)	357 (287, 454)	365 (224, 400)	0.169
N%, mean ± SD (median)	65.3 (53.3, 75.2)	64.9 (53.4, 74.6)	76.8 (55.9, 89.1)	0.026
NLR, mean ± SD (median)	2.4 (1.4, 4.1)	2.4 (1.4, 4.1)	3.5 (1.1, 11.7)	0.198
PLR, mean ± SD (median)	100.8 (69.2, 145.1)	100.4 (68.9, 144.1)	118.7 (71.0, 218.8)	0.158
TB, mean ± SD (median), mmol/L	5.1 (3.5, 7.7)	5.0 (3.5, 7.5)	7.9 (5.0, 11.2)	0.002

*SD* standard deviation, *CRP* c-reactive protein, *PLT* platelet count, *Na* serum sodium, *N%* percentage of neutrophils, *ALB* serum albumin, *ALT* alanine aminotransferase, *AST* aspartate aminotransferase, *TB* total bilirubin, *NLR* neutrophil-to-lymphocyte ratio, *PLR* platelet-to-lymphocyte ratio

^*^Fisher’s exact test was used

The scores of the prediction models were calculated for each patient, and then the patients were assigned to the high or low risk group. Table 3 shows the number of patients in the high and low-risk categories, together with the relative sensitivity, specificity, NPV, PPV and area of ROC curve of each model. Our prediction model had a sensitivity of 0.72 and a specificity of 0.75. Sensitivity of Kobayashi, Egami, Kawamura, Sano and Formosa were 0.72, 0.44, 0.48, 0.20, and 0.68, respectively. Specificity of these models were 0.62, 0.82, 0.66, 0.91, and 0.48, respectively.

**Table 3:** Prediction score models in patients with IVIG resistance

Prediction models	Category	Response to IVIG		Sensitivity	Specificity	PPV	NPV	Area under the ROC curve	95% CI
		Resistant	Responsive						
Kobayashi	High risk	18	184	0.72	0.62	0.09	0.98	0.74	0.65–0.84
	Low risk	7	295						
Egami	High risk	11	88	0.44	0.82	0.11	0.97	0.70	0.59–0.81
	Low risk	14	391						
Kawamura^a^	High risk	10	140	0.48	0.66	0.07	0.96	0.58	0.45–0.71
	Low risk	11	267						
Sano	High risk	5	43	0.20	0.91	0.10	0.96	0.58	0.46–0.70
	Low risk	20	436						
Formosa	High risk	17	248	0.68	0.48	0.06	0.97	0.58	0.48–0.68
	Low risk	8	231						
Ours	High risk	18	120	0.72	0.75	0.13	0.98	0.80	0.72–0.88
	Low risk	7	359						

*PPV* Positive predictive value, *NPV* Negative predictive value, *ROC* receiver operating characteristic, *CI* confidence interval

^a^76 patients were excluded for incomplete data when we calculated the Kawamura prediction model scores

## Discussion

In the present study, we evaluated the efficacy of the Kobayashi, Egami, Kawamura, Sano, Formosa, as well as our own prediction model using the medical data of KD in the past 2 years. We found Kobayashi and our prediction model had a relatively high sensitivity of 72% while our model had a higher specificity than Kobayashi. Sensitivity of Egami, Kawamura and Sano were all lower than 50%. Sensitivity of Formosa was intermediate with the lowest specificity. Our data suggested that our prediction model had a powerful predictive value in this area, followed by Kobayashi model while all the other prediction models had less excellent performances than ours. Actually, these findings might provide evidences for our future work on additional initial treatment in KD patients.

Kobayashi, Egami and Sano prediction models were mainly based on age, illness day and laboratory variables. The related predictive risk factors such as higher levels of CRP and N%, lower level of ALB were later proved to be effective predictors in different areas in our country [9, 11, 12]. However, the node and the point score in each prediction model were different due to a regional difference.

Developed in 2015, Formosa model was based on KD-associated manifestations besides laboratory characteristics. They developed the model in 181 patients and further validated the data in another 67 patients. The validation results showed the sensitivity and specificity of the prediction model were 71.4% and 81.0%, which was slightly lower compared with those in the development dataset [7]. Although two of the three predictors (N% and ALB) were also included in our prediction model, Formosa model didn’t have excellent performance in the present study. Our results were in consistence with Song’s study [13]. The assignment of 2 points to N% ≥60% in the Formosa model might increase the number of high-risk patients identified by that model.

Kawamura prediction model was also developed in Japan based on a single-center data. However, the prediction model was developed in 2016. That was to say, the prediction model was not fully established when we started the present study and thus we retrospectively reviewed the data according to the model at the end of our study. The prediction model was proved to have a sensitivity of 71% and a specificity of 69% in the combination of both neutrophil-to-lymphocyte ratio (NLR) ≥3.83 and platelet-to-lymphocyte ratio (PLR) ≥150 [8]. In another study including 437 KD patients which was carried out in the same medical center, the authors found the sensitivity and specificity of the prediction model were similar to Kobayashi model [14]. However, NLR and PLR could be calculated quickly and easily with the blood routine test alone. Neutrophils are the most abundant inflammatory cells, which may partly reflect the severity of the inflammation. In KD mouse models, influx of neutrophils and macrophages occurred 1 day after the stimulus, followed by a second neutrophil infiltration 28 days later, which persisted for several weeks [15]. In autopsied KD patients, neutrophils predominated in the earlier stage of the disease when macrophages predominated in the later stage [16]. The specific mechanisms remained obscure and were possibly related to the decreased apoptosis of neutrophils [17, 18]. Due to the activation of macrophages, elevated level of platelet was observed in most patients in the convalescent stage. On the other hand, lymphocytes also played a pivotal role in inflammatory processes and lymphocytopenia was associated with more severe inflammation owing to increased apoptosis [19, 20]. Although combination of NLR ≥3.83 and PLR ≥150 was more effective than either the NLR or PLR alone, an NLR of ≥3.83 or a PLR of ≥150 was an independent predictor of IVIG resistance [8]. Thus, patients with more than one predictor were assigned to high risk group and the remaining were assigned to low risk group. Unfortunately, the easily calculated prediction model had less powerful predictive efficacy. Factors such as different treatment regimens and methodology might be associated with the divergence.

It was not surprising that our prediction model had a higher efficacy in predicting IVIG resistance. We previously compared the clinical and laboratory characteristics of the patients with different IVIG responses. The sensitivity and specificity were 71.4% and 76.0% in the development dataset, respectively, which were similar to the results in the present study [9]. The divergences of sensitivity and specificity among these models and ours might be explained by different genetic backgrounds [1], definition of IVIG resistance and meteorological factors [21].

Though the incidences of IVIG resistance varied all of the world, mostly were between 10% and 20% [22–24]. It was quite low in the present study, which was similar to the incidence of Shanghai in the latest survey [25]. We previously had found that 5.1% of all patients were IVIG resistant during the past 9 years [9], which was almost the same as the present study. We speculated the time of initial IVIG treatment, racial differences and lots of IVIG might attribute to the discrepancy in the incidences. Unfortunately, all of these six prediction models had a relatively low PPV. Our results were coincident with previous study [13], which could be explained by a low IVIG resistance incidence (5% in the present study).

All the prediction models described in the present study were developed in Asia populations, most of which were from Japan. However, all had shown moderate sensitivity. What was more, previous study had found a lower sensitivity in an American population [26]. Due to an unknown origin of KD, we speculated a prediction model combined clinical, laboratory characteristics, with biomarkers or other specific indicators might have a better performance.

It should be noted that a total of 505 patients were diagnosed with KD in 2015 and 2016, which were almost half of those diagnosed between 2006 and 2014 [9]. In 2014, the Chinese government introduced the second child policy, leading to a dramatic increase in the number of children aged under 2 years in recent years. Thus, it was not surprising that the number of KD patients were increasing rapidly.

This study had some limitations. First, part of the study was retrospective and the missing data might lead to selection bias. Second, we did not conduct stringent validation by using an independent KD cohort from other institutions in this area, although most KD patients were diagnosed and treated in our hospital. Third, we didn’t evaluate the efficacy of other models such as Tremoulet prediction model for the inconvenience to get such variables. Further large-scaled studies were warranted to further verify the efficacy of the prediction models and moreover, to develop a more efficient prediction model suited for this area.

## Conclusion

Our prediction model had a powerful predictive value in this area, followed by Kobayashi model while all the other prediction models had less excellent performances than ours. The prediction model could help identify IVIG resistance in clinical work.

## Abbreviations

ALB: Serum albumin
ALT: Alanine aminotransferase
AST: Aspartate aminotransferase
CAA: Coronary artery abnormality
CRP: C-reaction protein
IVIG: Intravenous immunoglobulin
KD: Kawasaki disease
N%: Neutrophils proportion
Na: serum sodium
NLR: Neutrophil-to-lymphocyte ratio
NPV: Negative predictive value
PLR: Platelet-to-lymphocyte ratio
PLT: Platelet
PPV: Positive predictive value
ROC: Receiver operating characteristic
TB: Total bilirubin
WBC: White blood cell
